# Modified Cross-Interdental Wiring Technique for Intermaxillary Fixation

**DOI:** 10.7759/cureus.109037

**Published:** 2026-05-17

**Authors:** Nagasaireddy Konda, Divya Puvvada, Anil Budumuru, Manojkumar Musunuri, Rajasatish Prathigudupu

**Affiliations:** 1 Department of Oral and Maxillofacial Surgery, Sibar Institute of Dental Sciences, Guntur, IND; 2 Department of Oral and Maxillofacial Surgery, Vishnu Dental College, Bhimavaram, IND; 3 Department of Oral and Maxillofacial Surgery, Employees State Insurance Corporation (ESIC) Dental College, Kalaburagi, IND

**Keywords:** arch bars, circumdental wiring, intermaxillary fixation, mandibular fractures, occlusion

## Abstract

The management of maxillofacial fractures relies on fracture reduction, fixation, immobilisation, and restoration of occlusion. Intermaxillary fixation (IMF) is conventionally achieved using arch bars and circumdental wiring; however, these methods are time-consuming and may cause periodontal injury, particularly when only temporary intraoperative stabilisation is required. This study proposes a modified cross-interdental wiring technique for temporary IMF. The method utilises 26-gauge stainless steel wires passed obliquely between maxillary and mandibular molars to achieve buccolingual stabilisation, with twisted ends forming secure rosettes. The technique is indicated in minimally displaced fractures, orthognathic procedures, and paediatric cases where prolonged postoperative IMF is unnecessary. It is contraindicated in severely displaced fractures and edentulous patients. This approach is simple, minimally invasive, requires less operative time, and maintains adequate occlusion. Although challenges exist in compromised dentition and wire removal, the technique offers an efficient alternative to conventional IMF methods for intraoperative stabilisation.

## Introduction

The management of maxillofacial fractures is based on the fundamental principles of fracture reduction, fixation, and immobilisation, along with the restoration of normal occlusion to achieve optimal functional outcomes [1]. Conventionally, intermaxillary fixation (IMF) is achieved using wires to a hybrid or regular arch bar fixed to the maxillary and mandibular arches. In several clinical scenarios, however, IMF is required only temporarily during the intraoperative period and is not indicated postoperatively. Such situations include minimally displaced mandibular fractures managed with open reduction and internal fixation, intraoperative stabilisation during enucleation of jaw pathologies, or procedures where transient occlusal guidance is sufficient [2-4]. The Erich arch bar with circumdental wiring, although widely used, is time-consuming to apply and is associated with potential periodontal injury [3,5]. Moreover, placement of arch bars under general anaesthesia adds to operative time and may be unnecessary when prolonged postoperative IMF is not planned. In this context, the need exists for a simple, rapid, and minimally invasive technique that provides adequate intraoperative intermaxillary stabilisation without the drawbacks of conventional arch bars. We propose a modified temporary IMF technique using a cross-interdental wiring method that provides effective buccolingual stabilisation of the dental arches during surgery.

## Technical report

Technique

**Figure 1:**
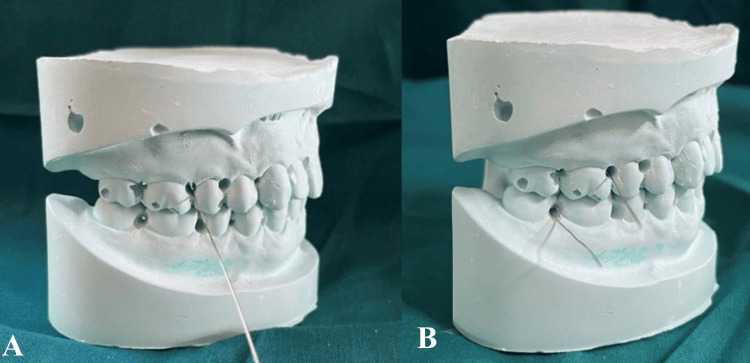
First wire passed from the maxilla to the mandible (A & B).

In this technique, pre-stretched 26-gauge round stainless-steel wires, approximately 12-15cm in length, are used. The first wire is passed through the mesio-buccal interdental embrasure of the maxillary first molar and directed obliquely towards the lingual aspect, exiting through the disto-buccal embrasure of the mandibular first molar (Figures 1A, 1B).

**Figure 2:**
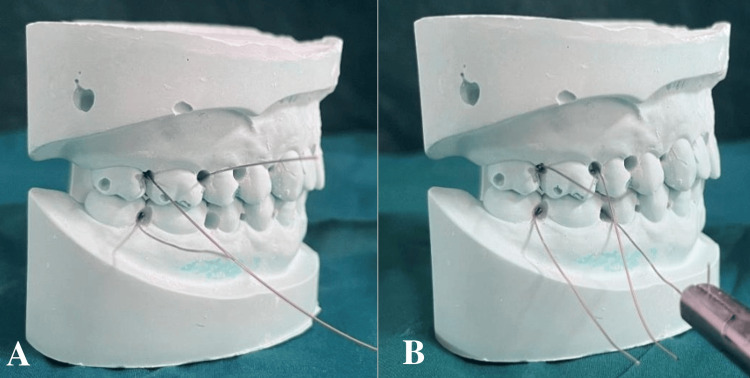
Second wire passed from the maxilla to the mandible (A & B).

Similarly, the second wire is passed from the disto-buccal side of the maxillary first molar, and it passes palatally obliquely through the lingual and exits through the mesio-buccal aspect of the mandibular first molar (Figures 2A, 2B).

**Figure 3:**
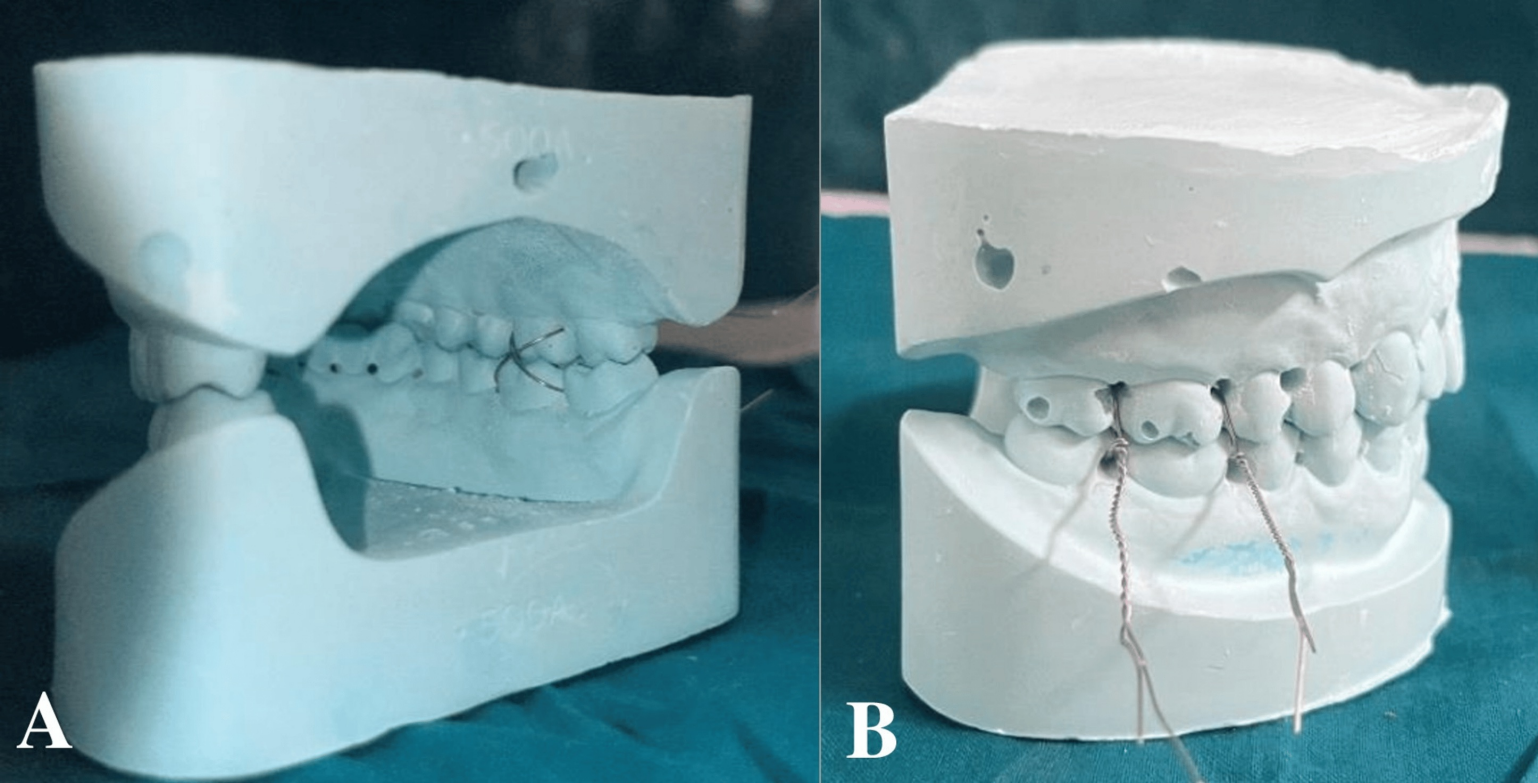
A, Lingual cross; B, Upper and lower wires on the mesial and distal sides are looped.

Then, both the distal ends of the maxilla and mandibular wires are twisted to form a rosette. Similarly, the mesial ends of the maxilla and mandibular wires are twisted to form another rosette (Figures 3A, 3B).

**Figure 4:**
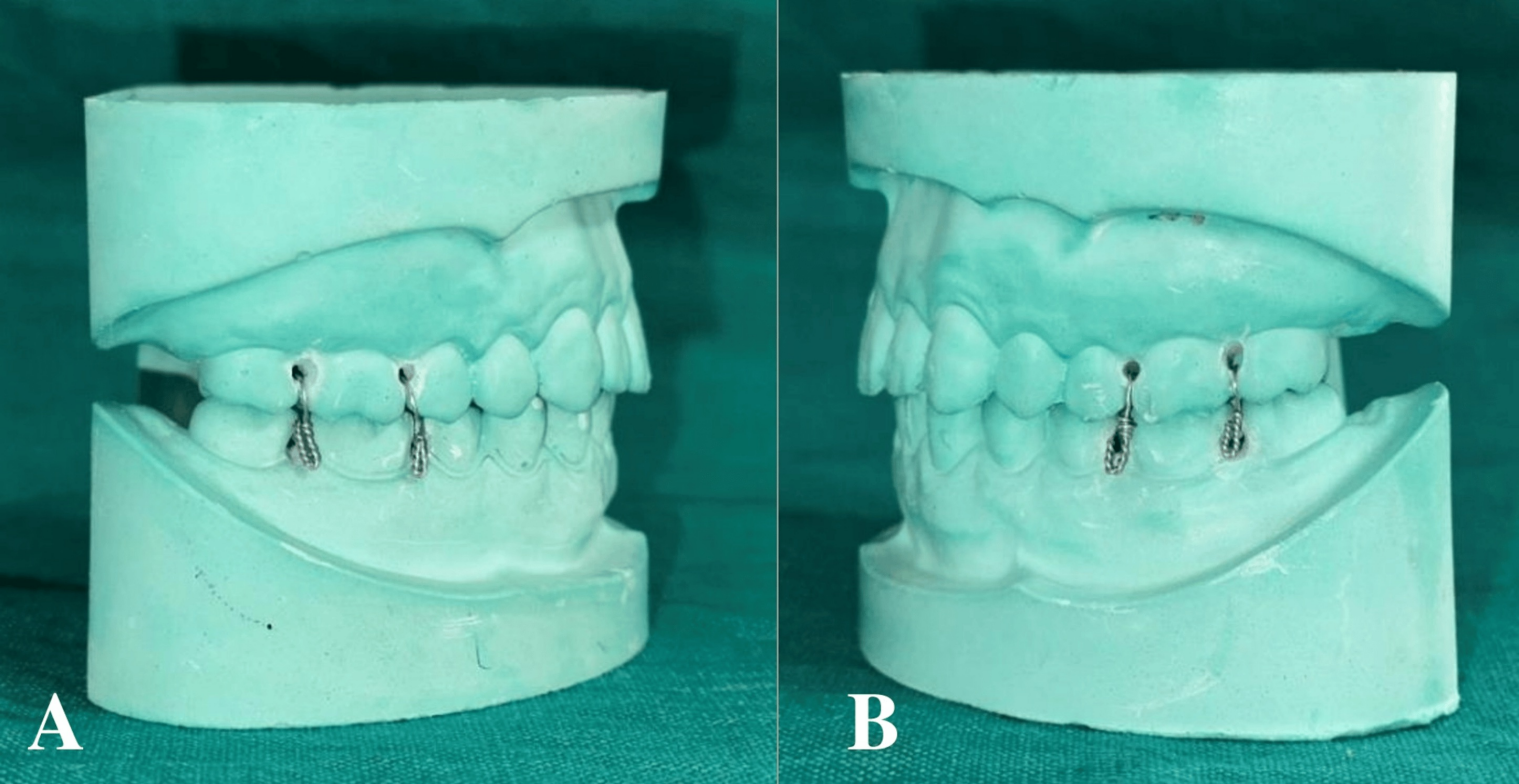
Both looped wires are twisted and tucked (A & B).

The ends of both rosettes are further twisted and tucked. Likewise, the same procedure is performed on the opposite side to achieve good, rigid IMF (Figures 4A, 4B).

It is indicated in patients with undisplaced mandibular fractures, where the bone segments remain stable and do not require rigid immobilisation. It is also suitable in orthognathic surgeries, where controlled occlusion and fixation are achieved intraoperatively, minimising the need for prolonged postoperative IMF. In paediatric mandibular fractures, this approach is preferred to avoid interference with jaw growth and to reduce patient discomfort. Additionally, it is beneficial in cases where early mobilisation and oral function are desired. Overall, it helps in maintaining occlusion while reducing complications associated with long-term IMF, such as poor oral hygiene and joint stiffness.

It is contraindicated in severely displaced mandibular fractures, where accurate reduction and stabilisation cannot be achieved without rigid fixation or prolonged immobilisation. It is also not suitable for partially or completely edentulous patients who lack stable posterior occlusal contacts, as adequate IMF cannot be maintained. Additionally, patients with periodontally compromised teeth are poor candidates, since weakened supporting structures may lead to tooth mobility or failure of fixation. In such cases, alternative methods of fracture management should be considered to ensure proper healing and functional stability.

This technique offers several advantages, as it is less technique-sensitive than conventional methods and can be performed with relative ease compared to more complex fixation methods. It provides a simpler and more efficient approach for achieving IMF, making it particularly useful in routine clinical settings. The procedure requires minimal armamentarium, reducing both cost and setup time. Additionally, it causes minimal trauma to the adjacent periodontal tissues, thereby preserving oral health and supporting structures. Overall, it is a time-saving method that enhances clinical efficiency while ensuring adequate stabilisation.

## Discussion

A successful IMF technique should be simple, inexpensive, minimally invasive, safe for the patient, secure the appropriate occlusion, and include an emergency quick-release system. Some authors have used single wires for IMF with bucco-lingual stabilisation [3,6,7] and double wire brace, as well as single wire brace techniques. In our clinical experience, we believe that the modified design addresses most of these desirable characteristics. It is an easy, simple, affordable, and minimally invasive procedure that firmly maintains the desired occlusion. No specific equipment or laboratory work is required. The main advantage is time saving, as it takes less than 10 minutes and can be released quickly in an emergency [3,7].

This technique has certain drawbacks, particularly in patients with multiple missing teeth or periodontally compromised dentition, where achieving stable IMF can be difficult. Additionally, it requires the use of two wires instead of a single wire, which may increase procedural complexity and material usage. Another limitation is that the removal of these wires can be time-consuming, potentially prolonging chairside time and causing inconvenience to both the clinician and the patient.

## Conclusions

The modified cross-interdental wiring technique offers a simple, cost-effective, and reliable method for achieving IMF. The method is easy to perform, requires minimal armamentarium, and demonstrates satisfactory clinical outcomes with good patient tolerance. Further studies with larger samples are needed to validate its long-term efficacy.
